# Effects of Kami Guibi-tang in patients with mild cognitive impairment: study protocol for a phase III, randomized, double-blind, and placebo-controlled trial

**DOI:** 10.1186/s12906-022-03805-9

**Published:** 2022-12-02

**Authors:** Hee-Yeon Shin, Tae-Bin Yim, Hye-Min Heo, Geon-Ho Jahng, Seungwon Kwon, Seung-Yeon Cho, Seong-Uk Park, Woo-Sang Jung, Sang-Kwan Moon, Chang-Nam Ko, Jung-Mi Park

**Affiliations:** 1 Department of Internal Korean Medicine, Korean Medicine Center, CHA university Bundang Medical Center, 59, Yatap-ro, Bundang-gu, Seongnam-si, Gyeonggi-do 13496 Republic of Korea; 2 Department of Clinical Korean Medicine, Graduate School, Kyung Hee University, 26 Kyungheedae-ro, Dongdaemun-gu, Seoul, 02447 Republic of Korea; 3 Stroke and Neurological Disorders Center, Kyung Hee University Hospital at Gangdong, 892, Dongnam-ro, Gangdong-gu, Seoul, 05278 Republic of Korea; 4 Department of Radiology, Kyung Hee University Hospital at Gangdong, College of Medicine, Kyung Hee University, 892, Dongnam-ro, Gangdong-gu, Seoul, 05278 Republic of Korea; 5 Department of Cardiology and Neurology, College of Korean Medicine, Kyung Hee University, 26 Kyungheedae-ro, Dongdaemun-gu, Seoul, 02447 Republic of Korea

**Keywords:** Mild cognitive impairment (MCI), Amnestic mild cognitive impairment (aMCI), Kami Guibi-tang, Herbal medicine, Herbal formula, Korean medicine, Neuropsychological test, Functional magnetic resonance imaging (fMRI)

## Abstract

**Background:**

Amnestic mild cognitive impairment (aMCI) is often considered a precursor to Alzheimer’s disease (AD) and represents a key therapeutic target for early intervention of AD. However, no treatments have been approved for MCI at present. Our previous pilot study has shown that Kami Guibi-tang (KGT), a traditional herbal prescription widely used in Korean medicine for treating amnesia, might be beneficial for improving general cognitive function of aMCI patients. We will conduct a larger-scale clinical trial to validate the findings of our pilot study and further examine the efficacy and safety of KGT in aMCI.

**Methods:**

This trial is designed as a randomized, double-blind, placebo-controlled clinical trial. A total of 84 aMCI patients will be recruited and randomized into the treatment and control groups. Participants will be administered either KGT or placebo granules for 24 weeks, with a follow-up period of 12 weeks after the last treatment. Primary outcomes will include changes in cognitive performance assessed using a neuropsychological test battery, called the Seoul Neuropsychological Screening Battery, between the baseline, post-intervention visit, and follow-up visit (24th and 36th week, respectively). Secondary outcomes will involve the rate of progression to AD, changes in neuroimaging signals assessed using structural magnetic resonance imaging (MRI), resting-state functional MRI (rs-fMRI), and task-based fMRI, and changes in blood biomarkers measured by the ratio of plasma amyloid-β 42/40 levels (Aβ42/Aβ40) between the baseline and post-intervention visit (24th week). For safety assessments, blood chemistry tests and electrocardiograms (ECG) will also be performed.

**Discussion:**

This study aims to provide confirmatory evidence of the effect of the Korean herbal medicine, KGT, on improving cognitive function in patients with aMCI. We will identify the possible mechanisms underlying the effects of KGT using neuroimaging signals and blood biomarkers.

**Trial registration:**

Korean Clinical Trial Registry (https://cris.nih.go.kr/cris/search/detailSearch.do/16918; Registration number: KCT0007039; Date of registration: February 24, 2022).

**Supplementary Information:**

The online version contains supplementary material available at 10.1186/s12906-022-03805-9.

## Background

Mild cognitive impairment (MCI) is a transitional stage between the cognitive changes in normal aging and those found in dementia. It is characterized by self- or informant-reported cognitive complaints and objective cognitive decline with preserved independence in daily abilities [1]. Up to 60% of patients with MCI develop dementia within a 10-year period [2]. The amnestic form of MCI, in which memory loss is the predominant symptom, is associated with the pathology of Alzheimer’s disease (AD) and is often a prodromal stage of AD [3]. About 10–15% of patients with amnestic mild cognitive impairment (aMCI) progress to AD every year, and almost 50% of these patients convert to AD after 3 years [4]. Early intervention at the MCI stage may delay the progression to AD [5]. However, there are currently no effective pharmacological interventions for MCI [6].

Kami Guibi-tang (KGT) (*Kamikihito* in Japanese) and Guibi-tang (*Kihito* in Japanese) are traditional herbal formulae commonly used in Kampo and Korean medicine for the treatment of amnesia, loss of appetite, depression, and insomnia. A few experiments and clinical studies have examined how KGT may affect cognitive function, especially in mild AD. KGT improved learning performance in senescence-accelerated mice model [7] and ameliorated impairment of spatial memory induced by scopolamine [8]. It also improved deficits in object recognition memory in 5XFAD AD transgenic mice model [9]. A cross-over clinical trial showed that the Mini-Mental State Examination (MMSE) scores of patients with AD increased significantly during kihito intake period [10].

Since there was no previous data for patients with MCI, we conducted a pilot trial to explore the potential effect of KGT in aMCI patients and examined the feasibility of performing a large-scale trial [11]. Thirty-three aMCI patients took either KGT or placebo granules for 24 weeks, and the general cognition as measured by the Clinical Dementia Rating-Sum of Boxes (CDR-SB) was significantly improved in KGT group compared to the placebo group. The intervention was well tolerated with no severe adverse events. We designed a larger scale randomized clinical trial to validate the findings of our pilot study and further investigate the efficacy and safety of KGT for enhancing cognitive function, including memory performance of aMCI patients. We will also examine the effect of KGT on the progression to AD and investigate the possible underlying mechanisms using neuroimaging modalities, including resting-state functional magnetic resonance imaging (rs-fMRI) and task-based fMRI, and amyloid blood biomarkers.

## Methods

### Study design

**Fig. 1:**
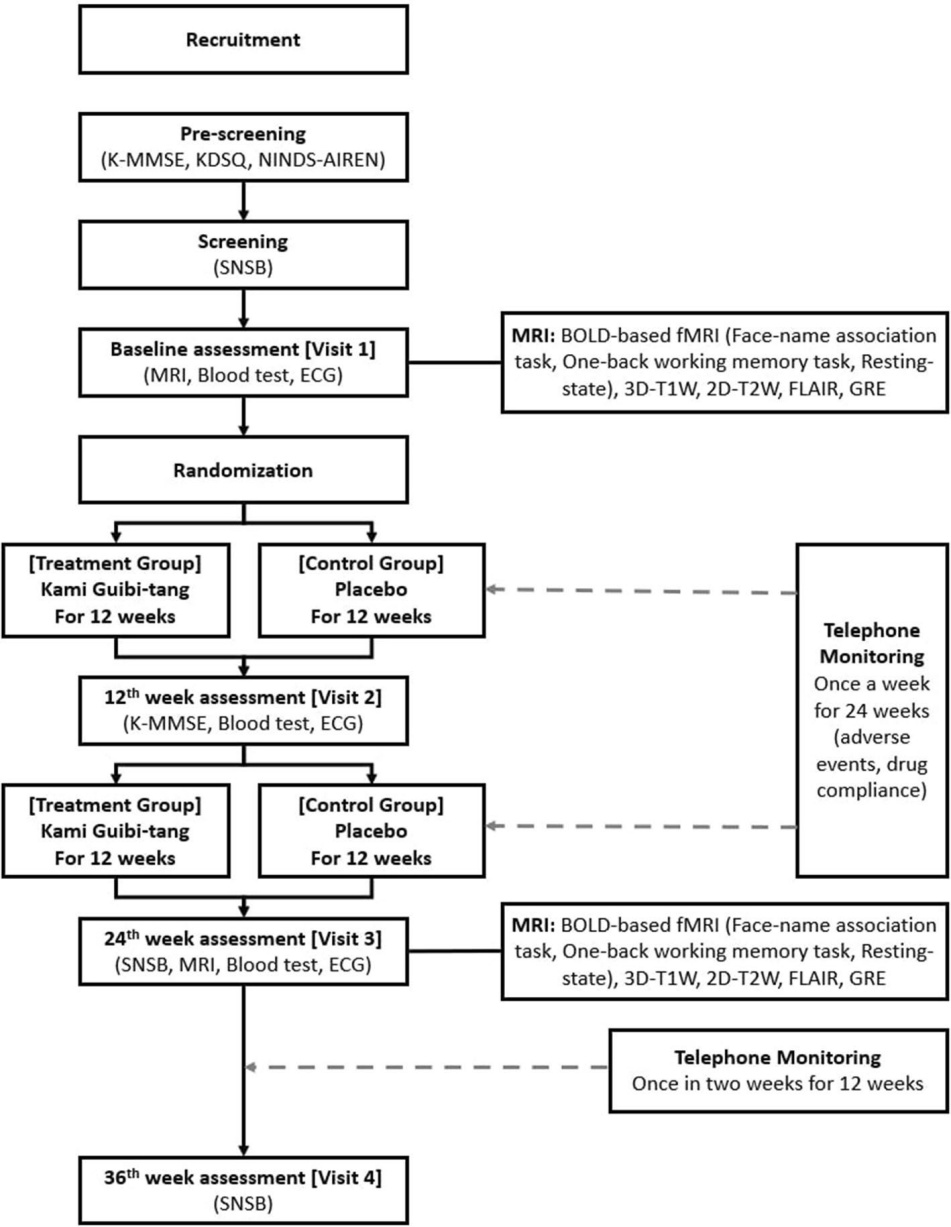
Flowchart of the trial design

This trial was designed based on our previous pilot study [11]. It will be a single-center, randomized, double-blind, placebo-controlled clinical trial with a 24-week intervention and a 36th week follow-up examination. It has been initiated at the Kyung Hee University Hospital at Gangdong, Seoul, Korea, in December 2021 and will be continued till May 2025. A flowchart of this trial is shown in Fig. 1. The schedules for enrollment, interventions, and assessments are presented in Table 1.

**Table 1:**
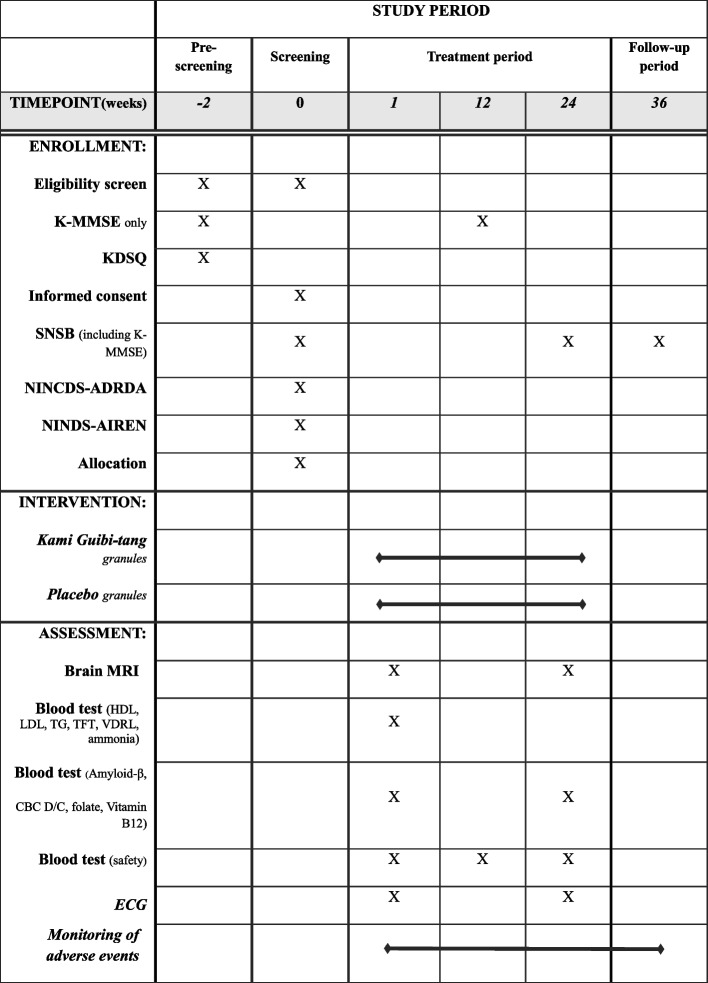
Schedule of enrollment, interventions, and assessments

### Participants

#### Inclusion criteria

Participants who meet the following criteria will be included: (1) adults aged 55–90 years, with complaints of impaired memory; (2) objective cognitive decline as measured using the Seoul Neuropsychological Screening Battery (SNSB), with a score of 0.5 on the Clinical Dementia Rating (CDR), 3 on the Global Deterioration Scale (GDS), and a normal score on the Korean MMSE (K-MMSE); (3) diagnosed with amnestic MCI by a neurologist; (4) not taking any medication that could affect cognitive function (e.g., gliatilin, gliatamine, ginexin, tanamin, or other psychoactive drugs) within 2 weeks; (4) no change in medication for underlying diseases within 2 weeks, and no expected change in medication during the trial; (5) no difficulty in communication.

#### Exclusion criteria

Participants who meet any of the following criteria will be excluded: (1) diagnosed with AD according to the criteria of the National Institute of Neurological and Communicative Disorders and Stroke-Alzheimer’s Disease and Related Disorders Association (NINCDS-ADRDA); (2) diagnosed with vascular dementia according to the criteria of the National Institute of Neurological Disorders and Stroke-Association Internationale pour la Recherche et l’Enseignement en Neurosciences (NINDS-AIREN); (3) with brain disorders that cause neurologic symptoms, other than cognitive impairment; (4) with other neurodegenerative disorders (e.g., Huntington’s disease, Parkinson’s disease, Creutzfeldt-Jakob disease and Down’s syndrome); (5) cognitive decline due to other diseases (e.g., vitamin deficiency, brain tumor, head trauma, encephalitis, hypoxic brain damage, neurosyphilis); (6) with history of major depressive disorder; (7) with psychiatric disorders or behavioral problems requiring antipsychotic medication; (8) with history of convulsive disorder, except for febrile convulsions during childhood; (9) with life-threatening or unstable medical conditions; (10) uncontrolled hypertension; (11) with heart or renal diseases; (12) with edema; (13) gastrointestinal symptoms (e.g., diarrhea, nausea, anorexia, abdominal pain); (14) use of medication that may induce myopathy or hypokalemia; (15) with hypersensitivity to ingredients in study medication; (16) abnormal range of liver and kidney function (serum aspartate aminotransferase/alanine aminotransferase (AST/ALT) levels more than two-fold the upper reference limit, or serum creatinine (Cr) levels more than 10% of the upper reference limit); (17) participating in other clinical trials within 4 weeks; (18) with illiteracy; (19) those who are contraindicated for Brain MRI; (20) any other possible conditions considered inappropriate to participate in the trial.

#### Drop-out criteria

Participants will be withdrawn from the trial if they meet the following criteria: (1) occurrence of any severe adverse events; (2) voluntary withdrawal from the trial; (3) not following the study protocol (i.e., drug compliance below 80%); (4) use of additional medication to improve cognitive function during the trial; (5) considered unsuitable by the principal investigator; (6) loss of ability to communicate and express their opinions.

### Enrollment

We plan to recruit a total of 84 amnestic MCI patients through physician referrals and newspaper advertisements. Candidates with memory complaints will be screened using the eligibility criteria, and the screening tools including the Korean Dementia *Screening* Questionnaire (KDSQ) and K-MMSE. SNSB will be conducted for potentially eligible participants, and those who are diagnosed with amnestic MCI will be enrolled. A clinical research coordinator and investigators will provide the participants with a detailed explanation of the study procedures, potential benefits and risks of participation. Informed consent will be obtained from all participants prior to enrollment.

### Allocation and blinding

An independent statistician will generate a random sequence using R Software version 4.1.3. All individuals will be randomly assigned to either KGT or placebo group, in a ratio of 1:1 using a block randomization with a block size of four. The random sequence will be sent to the pharmaceutical company as an encoded file, which will label and package the investigational drugs. Participants will be sequentially assigned and provided with the drug according to the randomization code in the order of enrollment. The participants, clinical psychologists, neuroimaging specialists, a clinical pharmacist, clinical research coordinator (CRC), clinical research associates (CRA), project manager (PM) of contract research organization (CRO), and study investigators will be blinded to treatment allocation until the completion of the study. Only if serious adverse events (SAEs) occur, the cases will be unblinded and the participants will be taken off trial.

### Intervention

The participants assigned to intervention group will take KGT granules (3 g /1pack) and those assigned to control group will take placebo granules (3 g /1pack). KGT is an herbal formula composed of 15 medicinal herbs. The amount and composition of each compound are presented in Table 2. *Dimocarpus Longan*, one of the herbal drugs of KGT, is a subtropical and tropical fruit of the Sapindaceae family. *D. longan* used in this trial was cultivated for medicinal purposes, not harvested from the wild. It is imported for medicinal use, and standardized, regulated, and quality controlled by the Korean Ministry of Food and Drug Safety (KMFDS) guidelines such as Korean Pharmacopoeia (KP) and Korean Herbal Pharmacopoeia (KHP). KGT will be manufactured by Han Poong Pharmaceutical Co., Ltd. (Jeonju, Korea), certified for Good Manufacturing Practices (GMP). Placebo drugs will be produced by the same manufacturer using the standard method of placebo manufacturing according to the Korean GMP guidelines. Their taste, color, shape, smell, and package will be made similar to KGT granules.

**Table 2:** Composition of Kami Guibi-tang

Scientific name	Latin name	Amount (g)
*Panax ginseng C.A. Meyer*	Ginseng Radix	1
*Atractylodes macrocephala Koidzumi*	Atractylodis Rhizoma Alba	1
*Poria cocos Wolf*	Poria Sclerotium	1
*Astragalus membranaceus Bunge*	Astragali Radix	1
*Dimocarpus longan Loureiro*	Longanae Arillus	1
*Zizyphus jujuba Miller var. spinosa Hu ex H.F. Chou*	Zizyphi Semen	1
*Bupleurum falcatum Linné*	Bupleuri Radix	1
*Angelica gigas Nakai*	Angelicae Gigantis Radix	0.67
*Polygala tenuifolia Willdenow*	Polygalae Radix	0.67
*Gardenia jasminoides Ellis*	Gardeniae Fructus	0.67
*Paeonia suffruticosa Andrews*	Moutan Cortex	0.67
*Zizyphus jujuba Miller var. inermis Rehder*	Zizyphi Fructus	0.67
*Aucklandia lappa Decne.*	Aucklandiae Radix	0.33
*Glycyrrhiza uralensis Fischer*	Glycyrrhizae Radix et Rhizoma	0.33
*Zingiber officinale Roscoe*	Zingiberis Rhizoma Recens	0.33

An independent clinical pharmacist will distribute the granules to participants in each group. Participants will be instructed to dissolve the granules in a glass of warm water and take the solution 30 minutes before or between meals, thrice daily. The investigators and CRC will make weekly reminder phone calls to the participants during the intervention period, in order to help them adhere to medication schedule. Participants will be allowed to take drugs for underlying medical conditions during the study period; however, they will not be allowed to take any medicine that may affect cognitive function. They will be required to report all the drugs taken during the study period. The name and dosage of the medication will be recorded in the case report form (CRF). Participants will be asked to return the leftover drugs at each study visit, and the number of returned drugs will be counted for assessing drug compliance. Those who take less than 80% of study medication will be withdrawn from the study.

### Outcome

The primary outcome includes changes in cognitive performance after the administration of KGT or placebo granules. SNSB, which is composed of various cognitive tests such as CDR, GDS, and MMSE will be conducted to assess the cognitive function, and changes in SNSB results will be analyzed. The secondary outcomes include the rate of progression to AD at the endpoint, according to NINCDS-ADRDA and CDR-GS score ≥ 1. In addition, we will measure neuroimaging signals and blood-based biomarkers. Structural MRI and functional MRI (fMRI) will be adopted to observe neuroimaging signal changes. The changes in gray matter volume will be measured using structural MRI. Also, Rs-fMRI and task-based fMRI will be performed to compare the changes in functional connectivity among working memory and default mode networks and to measure the changes in brain activation during the face–name association task and one-back working memory task. Levels of plasma Aβ42 and Aβ40 will be measured as blood-based biomarkers. The safety outcomes include electrocardiogram (ECG) and blood chemistry (Na, K, Cl, AST, ALT, BUN, Cr, glucose, LDH, CPK).

### Assessment

#### K-MMSE and SNSB

K-MMSE will be performed at the screening visit and at 12th week for evaluating the general cognitive status. It is one of the most widely used screening tests to estimate the cognitive functioning [12]. SNSB-II will be conducted at baseline, 24th week, and 36th week, to measure the effect of KGT on cognitive function. Changes in the mean SNSB scores will be analyzed as a primary outcome. SNSB is a neuropsychological test battery most commonly used in South Korea to estimate global cognitive function. The test is composed of various cognitive subtests that assess five domains: memory, attention, language, visuospatial function, and executive function. It also includes other related tests including CDR, K-MMSE, Korean-Instrumental Activities of Daily Living (K-IADL), Barthel-Activities of Daily Living (BADL), and Short-form Geriatric Depression Scale (SGDS) [13]. We will use a modified version of SNSB for dementia (SNSB-D), to obtain a global cognitive function (GCF) score, which is the sum of all the test scores of five domains. The maximum GCF score is 300 points: 150/300 (50%) for memory, 17/300 (6%) for attention, 27/300 (9%) for language, 36/300 (12%) for visuospatial function, and 70/300 (23%) for frontal/executive function [14]. The detailed contents of SNSB-D are presented in Table 3.

**Table 3:** Contents of Seoul Neuropsychological Battery for dementia (SNSB-D)

Domains (total score)	Subtests	Maximum points
Attention (17)	Digit span forward	9
	Digit span backward	8
Language & related function (27)	K-BNT	15
	Calculation	12
Visuospatial function (36)	RCFT copy	36
Memory (150)	Orientation	6
	SVLT free/delayed recall	48
	SVLT recognition	12
	RCFT free/delayed recall	72
	RCFT recognition	12
Frontal/Executive function (70)	Motor impersistence	3
	Contrasting program	3
	Go-no-go test	3
	Fist-edge-palm	3
	Luria loop	3
	Categoric word generation	20
	Phonemic word generation	15
	Stroop test-color reading	20
GCF score		300

*K-BNT* Korean short version of the Boston Naming Test, *RCFT* Rey Complex Figure Test, *SVLT* Seoul Verbal Learning Test, *GCF* Global cognitive function

#### MRI

Brain MRI scans will be conducted at baseline and 24th week using a 3.0 T MRI system (Philips Ingenia, Best, The Netherlands) to observe the neurophysiological changes. During the resting acquisition, participants will be asked to be in a supine position, with their head positioned in the 32-channel head coil, fixed by soft foam pads to minimize the head movement, and to remain awake with their eyes closed. Task-based fMRI data will be obtained while participants perform a face–name association task and one-back working memory task. Blood oxygenation level-dependent (BOLD) data will be acquired using a T2-weighted gradient echo, echo-planar imaging (EPI) sequence with the following acquisition parameters: repetition time (T_R_) = 2000 ms; echo time (T_E_) = 30 ms; flip angle (FA) = 70°; field of view (FOV) = 210 × 210 × 96 mm, matrix = 70 × 70; number of slices = 32; slice thickness = 3 mm; slice gap = 0 mm; multi-band factor = 2; SENSE factor = 2; voxels = 3 × 3 × 4 mm^3^. A sagittal structural 3D T1-weighted (3D T1W) image will be acquired before and after contrast injection with a turbo fast field echo sequence, similar to the magnetization-prepared rapid acquisition of the gradient echo (MPRAGE) sequence with the following acquisition parameters: repetition time (T_R_) = 8.1 ms, echo time (T_E_) = 3.7 ms, flip angle (FA) = 8°, field-of-view (FOV) = 236 × 236 mm^2^, and voxel size = 1 × 1 × 1 mm^3.^

#### Blood testing

Plasma Aβ42 and Aβ40 levels will be measured at baseline and at 24th week to examine the disease-modifying effects, since the ratio of Aβ42/Aβ40 reflects the AD pathology [15, 16] and associated neurodegeneration, and is related to cognitive impairment [17]. Changes in the ratio of plasma Aβ42/Aβ40 will be compared between the groups. A ratio less than 0.1218 is defined as positive amyloidosis; therefore, the conversion rate to a positive Aβ42/Aβ40 ratio will also be compared. CBC D/C, folate, and vitamin B12 levels will be measured at baseline and 24th week. HDL, LDL, TG, TFT, VDRL, and ammonia levels will be assessed at 1st week.

#### Safety assessment

ECG and blood tests will be conducted to examine the safety outcomes. ECG will be conducted at baseline and 24th week. Blood levels of Na, K, Cl, AST, ALT, BUN, Cr, glucose, LDH, CPK will be measured at baseline, 12th, and 24th week. Vital signs will be checked at each visit. We will closely monitor any abnormal findings in vital signs, blood tests, and ECG.

### Adverse events (AEs)

Adverse events will be monitored at each visit, and by telephone, weekly during the intervention and biweekly for 12 weeks after the intervention. All AEs will be assessed for causality and severity, and the details of each AE will be recorded in CRF. If a serious adverse event (SAE) occurs, the participation will be terminated, and the institutional review board (IRB) will be reported as soon as possible. All AEs will be monitored until they stabilize.

### Data management and monitoring

Data will be entered into the electronic case record form (eCRF) by CRC and the investigators. The entered data will be monitored by CRAs, through the process of source data validation (SDV). Only the monitoring agent and the investigators will have access to the dataset. The eCRF files will be stored in the password-secured database called MyTrial. All procedures will comply with the confidentiality standards for medical data. All documents and collected data will be kept for 3 years after completion of the study, after which they will be destroyed. The data will be managed by a data management project manager (DMPM) and a data management associate (DMA), according to standard operating procedures (SOPs). The trial will be monitored by a monitoring committee which is composed of PM, CRA and clinical research manager (CRM). Auditing will be conducted by the Korean Ministry of Food and Drug Safety and IRB.

### Statistics

#### Sample size calculation

The primary outcome of interest in this study is the change in the total SNSB-D score between baseline and post-intervention. The calculation of sample size is based on a superiority test, as the aim of this study is to show that KGT is superior to placebo. Our pilot study of aMCI patients treated with KGT showed that the change in the total SNSB-D score was + 22.47, compared to + 8.36 in the placebo group. The calculated effect size (Cohen’s d) was 0.70. Using G*Power software with a power of 0.80 and a two-sided type I error of 0.05, the calculated total sample size for the current study involves 66 participants, with 33 in each group. The dropout rate was 10% (3 of 33 participants) in the previous study; however, since the current study will take a longer follow-up period, we expect the dropout rate to be 20%. We plan to recruit a total of 84 participants for this RCT (42 participants in each group).

#### Data analyses

An independent professional statistician, who will also be blinded to the allocation, will perform data analyses using SPSS 20.0 software (IBM, Armonk, NY, USA). Efficacy analyses will be performed using the modified intention-to-treat (mITT) and per-protocol (PP) principles. The mITT population will include participants who have completed at least one examination since the beginning of the intervention. The PP population will include participants who will adhere to the trial protocol and undergo all examinations. Missing data will be adjusted using the last-observation-carried-forward (LOCF) imputation method. A safety test will be performed using the ITT principle. The ITT population will include any participants who will take at least one pack of drugs after randomization. To analyze the efficacy outcome and continuous demographic variables, we will perform the Shapiro-Wilk test to confirm the normality of the data distribution. To analyze the fMRI measures and plasma amyloid beta levels, the paired *t*-test will be used for intra-group comparisons and an independent *t*-test will be used for intergroup comparisons, for normally distributed data. Wilcoxon’s signed rank test will be used for intra-group comparisons and the Mann-Whitney U test for intergroup comparisons of non-normally distributed data. To analyze the scores of the neuropsychological test battery, repeated measures of analysis of variance will be used for parametric data, and the Friedman test for non-parametric data. Categorical demographic variables, conversion to normal cognition or dementia, conversion to positive plasma Aβ42/Aβ40 ratio (< 0.1218), and the incidence of AEs between the groups will be analyzed using the Pearson’s chi-square (χ^2^) test or Fisher’s exact test. Continuous demographic variables will be analyzed using the *t*-test or Wilcoxon signed-rank test. All statistical analyses will be two-tailed, and the significance level will be set at *p* < 0.05.

## Discussion

This trial was designed based on our previous pilot study. To the best of our knowledge, it is the first confirmatory randomized clinical trial to evaluate the effect of KGT on cognitive function in aMCI patients.

We examined the feasibility of the trial process and the acceptability of KGT treatment in our previous study. Since the results showed a significant improvement in the CDR-SB score in the KGT group, the same neuropsychological test battery, including CDR, was selected as the primary outcome. Moreover, we observed high adherence and drug compliance, with 10% dropout rate. No serious safety concerns were reported.

Several improvements were considered in the planning of the current trial. First, as with most pilot studies, the relatively small sample size limited the statistical power and generalizability. Therefore, we will include a larger sample size in this trial, as calculated based on the results of our pilot trial. Second, there was no follow-up examination of cognitive function after completing the intervention in our previous trial, so the long-term effects of KGT could not be examined. Therefore, we will conduct a follow-up neuropsychological test 12 weeks after completing the treatment and observe any occurrence of AEs for 12 weeks after the treatment, compared to 4 weeks of post-intervention monitoring in the prior study.

The advantages of this trial include using extensive assessment tools to evaluate the outcomes. First, as in the pilot study, we will use SNSB, which is the full neuropsychological battery, which is composed of comprehensive and diverse cognitive tests, in order to sensitively capture the changes in cognitive function [13]. This is because the instruments frequently used in previous studies, such as MMSE and Alzheimer’s Disease Assessment Scale-Cognitive subscale, lack sensitivity in detecting mild degrees of cognitive decline, which lead to ceiling effects [18].

Next, we will perform multimodal MRI scans in this study, including structural MRI and resting-state and task-evoked functional MRIs. Structural MRI scan of brain atrophy are the most widely accepted markers of AD progression [19]. A decline in hippocampal volume has consistently been related to memory loss in the AD continuum [20]. Since fMRI, which is an indirect measure of brain activity, is useful in the detecting early alterations in brain function, it might be valuable for evaluating the physiological changes over a short time interval [21]. We will investigate the effects of KGT on brain function using both task-based and task-free (resting-state) approaches. Rs-fMRI studies have shown that the progression of memory decline in aMCI is associated with a decline in functional connectivity in DMN regions, which include brain regions related to cognitive function, such as the hippocampus and medial frontal lobe [22]. For task-based fMRI, we will adopt two types of memory paradigms: the face–name association task and the one-back working memory task. By employing cognitive tasks, the fMRI measure will provide information on the changes in brain activity while performing specific activities that mimic the actual difficulties occurring in daily life [21]. Various neuroimaging modalities will be helpful for evaluating the neural underpinning of the effect of KGT.

We will also observe changes in blood-based neuropathological biomarkers, including the plasma levels of Aβ42, which are proposed to reflect the AD pathology and associated neurodegeneration [15–17]. Plasma Aβ levels are correlated with the cerebrospinal fluid (CSF) Aβ levels and Aβ deposition in the brain [23]. Low plasma Aβ42/40 ratios are associated with an increased risk of dementia [24] and greater cognitive decline [25]. We will investigate whether KGT shows any disease-modifying effect via the plasma Aβ42/40 ratio, instead of using CSF biomarkers, which is a costly and invasive process. In addition, plasma Aβ protein levels may provide information about the etiology of MCI.

Despite the strengths of our study, there are some limitations to our protocol. Although we plan to conduct follow-up examinations at the 36th week, the treatment and follow-up periods are relatively short considering the nature of the disease, which is inevitable due to the limited study period. Nevertheless, the 24-week intervention indicated an improvement in cognitive function in our pilot study, and this issue could be resolved by incorporating fMRI measures that can help detect even subtle changes over a short period of treatment. Next, the signals that will be used in this trial, especially fMRI, do not yet provide a reliable AD biomarker or tracking tool due to inconsistent findings of previous studies. However, we anticipate that using a combination of multimodal MRI scans, blood biomarkers, and extensive neuropsychological tests may aid in the better understanding of the effects of treatment.

In summary, the findings of this trial will provide confirmatory evidence for the therapeutic effects of KGT in patients with aMCI. This study will also give insights into the mechanisms underlying the effects of KGT via various biomarkers.

## Trial status

This study began patient recruitment on February 4th, 2022.

## Supplementary Information

**Additional file 1.** The SPIRIT 2013 checklist.

**Additional file 2.** Consent Form (English version).

## Data Availability

Not applicable.

## Abbreviations

aMCI: Amnestic mild cognitive impairment
AD: Alzheimer’s disease
KGT: Kami Guibi-tang
SNSB: Seoul Neuropsychological Screening Battery
MRI: Magnetic resonance imaging
rs-fMRI: Resting-state functional MRI
Aβ: Amyloid-β
ECG: Electrocardiogram
MMSE: Mini-Mental State Examination
CDR-SB: Clinical Dementia Rating-Sum of Boxes
GDS: Global Deterioration Scale
CDR: Clinical Dementia Rating
K-MMSE: Korean Version of Mini-Mental State Examination
NINCDS-ADRDA: National Institute of Neurological and Communicative Disorders and Stroke-Alzheimer’s Disease and Related Disorders Association
NINDS-AIREN: National Institute of Neurological Disorders and Stroke-Association Internationale pour la Recherche et l’Enseignement en Neurosciences
AST: Aspartate aminotransferase
ALT: Alanine aminotransferase
Cr: Creatinine
KDSQ: Korean Dementia Screening Questionnaire
SAE: Serious adverse event
GMP: Good Manufacturing Practice
CRF: Case record form
CDR-GS: Clinical Dementia Rating-Global Score
fMRI: Functional MRI
BUN: Blood urea nitrogen
Na: Sodium
K: Potassium
Cl: Chloride
LDH: Lactate dehydrogenase
CPK: Creatine phosphokinase
BADL: Barthel-Activities of Daily Living
SGDS: Short-form Geriatric Depression Scale
SNSB-D: SNSB for dementia
GCF: Global cognitive function
BOLD: Blood oxygenation level-dependent
EPI: Echo-planar imaging
T_R_: Repetition time
T_E_: Echo time
FA: Flip angle
FOV: Field of view
3D T1W: Structural 3D T1-weighted
MPRAGE: Magnetization-prepared rapid acquisition of the gradient echo
CBC D/C: Complete blood count & differential count
HDL: High-density lipoprotein
LDL: Low-density lipoprotein
TG: Triglyceride
TFT: Thyroid Function Test
VDRL: Venereal Disease Research Laboratory
AE: Adverse event
IRB: Institutional review board
mITT: Modified intention-to-treat
PP: Per-protocol
LOCF: Last-observation-carried-forward
ITT: Intention-to-treat
ADAS-cog: Alzheimer’s Disease Assessment Scale-Cognitive subscale
CSF: Cerebrospinal fluid
