# Supplementary material for: Effects of Kami Guibi-tang in patients with mild cognitive impairment: study protocol for a phase III, randomized, double-blind, and placebo-controlled trial
Source: BMC Complement Med Ther. 2022 Dec 2;22:318. doi: 10.1186/s12906-022-03805-9 (PMC9717542; doi:10.1186/s12906-022-03805-9)
Supplement: Supplementary file 2 — Additional file 2. Consent Form (English version). [file 12906_2022_3805_MOESM2_ESM.docx]

**Information Sheet and Informed Consent Form (ICF)** version no. *1.4*

| **Study title** | The effect of Kami Guibi-tang for improving cognitive function in mild cognitive impairment patients : A randomized, double-blind, placebo-controlled Trial | | | |
| --- | --- | --- | --- | --- |
|  | **Department** | **Title** | **Name** | **Phone** |
| **Principal investigator** | Korean Medicine | Professor | Jung-Mi Park | 02-440-6216 |
| **Coinvestigator** | Korean Medicine | Professor | Chang-Nam Ko | 02-440-6215 |
| **Coinvestigator** | Korean Medicine | Professor | Seong-Uk Park | 02-440-6217 |
| **Coinvestigator** | Korean Medicine | Associate professor | Seung-Yeon Cho | 02-440-6209 |
| **Coinvestigator** | Korean Medicine | Resident | Gyu-Ri Jeon | 02-440-8557 |
| **Coinvestigator** | Korean Medicine | Resident | Hye-Jin Lee | 02-440-8557 |
| **Coinvestigator** | Korean Medicine | Resident | Ye-Chae Hwang | 02-440-8557 |
| **Investigator** | Korean Medicine | Resident | Tae-Bin Yim | 02-440-8557 |

**※ Please contact the above investigators in the case that you experience risks or discomfort as listed in the consent form or any other complications that had not been anticipated.**

Before you decide on participating in this clinical study, it is important to accurately understand why this clinical study is being conducted and what will be implemented and how in the study. The following has been prepared to give an explanation on the content of this study.

This study will be conducted only on subjects that express their voluntary willingness to take part. Please take your time to thoroughly read the following. If you wish, you may consult with other people, including your family. You are also welcome to ask questions you may have to the principal investigator or investigator. Your signature indicates that you have been given an explanation on this study and that you (or your legal guardian) wish for you to participate in this study.

**1. This clinical study is conducted for research purposes.**

This is a clinical study to compare and evaluate the safety and efficacy of Kami Guibi-tang in improving cognitive function in patients with mild cognitive impairment.

**2. Background, purpose, methodology, and anticipated effects of the clinical study**

One of the early symptoms of undermined cognition is MCI, or mild cognitive impairment. About 80% of MCI patients are reported to see their symptoms progress to dementia within one year, but to date there is no safe and effective drug for the treatment of MCI.

This clinical study will be conducted to confirm the rationale for Kami Guibi-tang to be used for a wider range of indications including MCI, as the safety and efficacy of Kami Guibi-tang have already been verified in preliminary clinical studies.

**1) Treatment method**

All patients participating in the study will make a total of four visits to the hospital as follows:

1) Before any administration of the test drug

2) At twelve weeks following the first administration of the test drug

3) After 24 weeks of administering the test drug

4) At twelve weeks following the administration of the test drug for 24 weeks

Blood tests and ECG tests will be conducted (before taking the test drug, after 12 weeks of taking the drug, and after the administration of the drug is completed (after 24 weeks of taking the drug).

A simple questionnaire (K-MMSE) will be conducted (before taking the test drug and twelve weeks following the first administration of the test drug).

An MRI test will be conducted (before taking the test drug and after 24 weeks of administering the test drug).

An SNSB test will be conducted (before taking the test drug, after 24 weeks of administering the test drug and after twelve weeks following the last administration of the test drug).

After an observation period over a total of 36 weeks, the questionnaire you fill out, along with your electronic medical records (detailing your blood test results, MRI test and SNSB test results) will be compiled for analysis.

A detailed explanation will be given by the investigator to both the control group and the experiment group regarding the tests and questionnaire results. Proactive medical evaluations will be carried out during the study period to manage the patients’ status.

**2) Efficacy and Effects**

Numerous studies have found Kami Guibi-tang to protect neurons, improve the deterioration of the nerves and significantly improve the memory of object recognition without side effects. Preliminary clinical studies, too, found it to be effective in improving cognitive function. As such, Kami Guibi-tang that the study subjects will be taking as part of this study is expected to improve the cognitive disorders in patients diagnosed with mild cognitive impairment and delay the progression of the illness.

**3) Study Subjects**

This study will be conducted on those who have been diagnosed with mild cognitive impairment by a neurologist who has not undergone any drug treatment in the last two weeks related to cognitive function, who has maintained a stable state without any changes to the drugs taken for any underlying illnesses, who do not fall within the category of disqualification based on imaging tests, and who can undergo treatment using Korean medicine.

**3. Drugs used in the study and the probability of being randomly assigned to the experiment group or the control group**

Kami Guibi-tang is a Korean medicine mostly prescribed for forgetfulness or insomnia. It has been reported to protect neurons, prevent neurological deterioration and significantly improve the memory of object recognition. It has also been found in a clinical study conducted in Japan to effectively improve cognitive function. In a preliminary clinical study conducted in Korea on patients with mild cognitive impairment with loss of memory, subjects reported seeing improved cognitive function overall after taking Kami Guibi-tang.

Subjects in the clinical study will be randomly assigned to the experiment group (who will take Kami Guibi-tang) or the control group (who will be taking a placebo). Those in the experiment group will take Kami Guibi-tang three times a day for a total of 24 weeks, while the control group will take a placebo three times a day for a total of 24 weeks.

As the clinical study subjects will be randomly assigned to either the control group or the control group, the probability of being assigned to either is 50%. Random assignments are done to prevent the willpower or intention of the investigators from intervening in the determination of the treatment method for a given patient. (It is comparable to assignments based on heads or tails following the toss of a coin).

**4. Tests and procedures that study subjects will undergo**

Clinical study subjects who grant their consent to participation in this study visit the hospital on a total of four occasions as follows:

1) Before taking the test drug,

2) At 12 weeks following the first administration of the test drug,

3) After 24 weeks of administering the test drug,

4) At 12 weeks following the administration of the test drug for 24 weeks.

The following tests or survey will be conducted:

Blood tests and ECG tests (before taking the test drug, after 12 weeks of taking the drug, and after the administration of the drug is completed (after 24 weeks of taking the drug).

A simple questionnaire (K-MMSE) (before taking the test drug and at 12 weeks following the first administration of the test drug).

An MRI test (before taking the test drug and after 24 weeks of administering the test drug).

An SNSB test (before taking the test drug, after 24 weeks of administering the test drug and at 12 weeks following the first administration of the test drug).

**5. Regulations that the study participant must follow**

Study subjects must take the assigned drug and undergo tests during the study period in accordance with the study plan, and shall be prohibited from taking part in any other clinical studies. Subjects are also required to report to the investigator on any changes that occur during the study participation period.

**6. Experimental aspects of the clinical study that are not verified**

The test drug used in this clinical study (i.e. Kami Guibi-tang) has been reported in existing experimental studies and preliminary studies as being effective in improving cognitive function, but is not a drug that has mild cognitive impairment listed as one of its indications.

**7. Anticipated risks (i.e. side effects) or discomfort as a result of participating in the clinical study**

As part of this clinical study, the SNSB test, blood test, and MRI test will be conducted. SNSB is a questionnaire used to diagnose and evaluate cognitive disorders and has no associated risks associated. Blood tests will be basic blood tests to evaluate any side effects from the drug and the ratio of Amyloid Beta at 42/40. The significant risks reported in association with this blood test are negligible. The MRI test also has negligible risks unless there are factors that would make the patient ineligible for imaging tests. If such ineligibility applies, the participant shall be excluded from the MRI test based on the judgment of the medical staff.

No side effects from Kami Guibi-tang were reported in preceding studies. In addition, mild cognitive impairment is usually a stage where drug therapy is either not conducted or is just started. As such, there is no risk of delayed treatment for the study subjects due to their assignment to the control group. Kami Guibi-tang and the placebo will both be supplied from Hanpoong Pharmaceutical Limited. There are no significant side effects associated with them that have been reported, either. The general precautions required in association with the drugs provided are as follows.

| 1. If you fall under any of the following categories, do not take this drug. Women who are pregnant or who may become pregnant. (The skin of the roots of Paeonia suffruticosa Andrews contained in the drug may pose a risk of early termination or stillbirth.)  2. If you fall under any of the following categories, please consult with your doctor, oriental medicine doctor, dentist, pharmacist or oriental pharmacist.  1) Patients suffering from hypertension  2) Those with diseases affecting the heart or kidneys.  3) Those suffering from edema  4) Those with advanced age (In general, those in the older age group have undermined physiological functions and therefore precautions must be taken, such as reducing the dosage.)  5) Patients who are being administered with other drugs as part of treatment  6) Those who have experienced rashes, reddening of the skin or itchiness caused by drugs.  7) Those with a weakened gastrointestinal system. (Such individuals may experience sluggish appetite, unpleasant feelings in the gastrointestinal system, nausea, stomach ache or diarrhea.)  8) Those with sluggish appetite or nausea. (Such individuals may see a worsening of their symptoms.)  3. In the following cases, immediately stop taking the drug and consult with your doctor, oriental medicine doctor, dentist, pharmacist or oriental pharmacist. If possible, bring this document with you for your consultation.  1) If the following symptoms have occurred as a result of taking this drug  (1) Pseudo-aldosteronism: The quantity of urine has decreased. The face and limbs are swollen. Eyelids feel heavier. Hands feel stiff. Blood pressure has increased or there is headache. (If drugs that involve a maximum intake of Glycyrrhiza uralensis Fischer of 1g or greater per day are taken for an extended period, pseudo-aldosteronism may occur, accompanied with low potassium levels in the blood, hypertension, retention of sodium in the body fluids, edema and weight gain. Sufficient observation (such as measurement of potassium levels in the blood) must be carried out. If abnormalities are confirmed, the intake of the drug must be suspended.)  (2) Myopathy: Myopathy may appear as a result of low potassium levels in the blood. Sufficient observation must be carried out. If lethargy, muscle cramps in the limbs or paralysis are confirmed, the intake of the drug must be suspended.  (3) Skin issues: Rashes, reddening of the skin, itchiness, hives  (4) Gastrointestinal issues: Loss of appetite, discomfort in the gastrointestinal system, nausea, diarrhea or stomach ache  2) If there is no easing of the symptoms even after taking the drug for about a month.  4. Other precautions to be taken when taking this drug  1) In principle, the drug should not be taken for an extended period. But if an extended intake is inevitable, consult with your doctor, oriental medicine doctor, dentist, pharmacist or oriental pharmacist.  2) Abide by the correct dosage.  3) Myopathy may occur due to pseudo-aldosteronism or low potassium levels in the blood if the drug is taken together with other drugs containing potassium, Glycyrrhiza uralensis Fischer, Glycyrrhizic acid or its derivatives, loop diuretics (Furosemide and Ethacrynic Acid) or thiazide diuretics (Trichlormethiazide).  4) When taking the drug with other oriental medicine, take precaution against taking the same raw ingredients contained.  5) Taking the drug may affect the test results on diabetes.  5. Precaution required for the storage of the drug  1) Store in a place away from direct sunlight and high humidity. Keep in a cool place. After use, store it completely sealed.  2) Store away from the reach of children. |
| --- |

**8. Anticipated benefits from participating in the clinical study**

The SNSB tests, ECG tests, blood tests and MRI tests will be conducted at the expense of the investigator. Subjects may request a detailed explanation from the investigator on the test results and questionnaire conducted.

**9. Other benefits offered to clinical study subjects**

Study subjects will be offered 100,000 won per hospital visit for travel expenses. The Kami Guibi-tang used for the experiment group and the placebo used for the control group will be provided at the expense of the investigator.

**10. Other treatments that may be considered for the same illness and the potential risks and benefits of such treatments**

Symptoms of cognitive disorder may improve with the administration of the drug used in the clinical study, but there may not be any marginal benefits or any benefits at all. However, as of now there is no safe and effective drug that can be used for the treatment of mild cognitive impairment.

**11. Expected participation period, the total number of study subjects and the number of study subjects from our hospital**

This clinical study is scheduled to take place until May 31, 2025 with a total of 66 patients who have been diagnosed with mild cognitive impairment. (A total of 84 people will be recruited to take into account the dropout rate). If you decide to participate in the clinical study, you will undergo a screening process that consist of an interview, tests and consultation with a doctor. Only those meeting the selection criteria will be selected.

**12. Compensation or treatment to be offered to study subjects in the case of an impairment or injury associated with the clinical study**

Medical staff will provide compensation and damages for impairment or injury directly incurred by the clinical study subjects as a result of having taken part in the clinical study, in accordance with the Terms and Conditions for the Compensation of Participants in the Clinical Study.

**13. Whether monetary compensation is offered in exchange for participation in the clinical study, the degree of adjustments made in accordance with the degree of participation and additional expenses expected to be incurred by the study participant as a result of participating in the clinical study**

All expenses related to the Kami Guibi-tang or placebo to be offered to the study subjects, questionnaires conducted for the clinical study, blood tests, all oriental medicine and tests shall be borne by the investigator. However, additional tests for a medical consultation on the neurological system that are not included in this study (e.g. amyloid PET) shall be paid for by the study participant.

**14. Timely notification to the study participant or his/her representative of new information that may affect the participant’s willingness to continue to participate in the clinical study**

**15. The study participant acknowledges that if deemed necessary by the principal investigator, the participant may be excluded from the study without the consent of the participant. In such a case, withdrawing from the study may be done by notifying the investigator or the principal investigator listed on the consent form.**

**16. Granting or withdrawal of consent based on free will**

The decision on whether to participate in the clinical study shall be made voluntarily. A study participant may withdraw his/her participation at any point during the study without losing any advantages that had been earmarked for him/her. You have the freedom not to participate in this study. Non-participation will not disadvantage you in any way in your relationship with this hospital or its doctors.

**17. Use of personal data and confidentiality**

All your records will be kept confidential, even in cases where the findings from the clinical study are published. However, they may be disclosed to those associated with this study, for research purposes. Even in such a case, your personal data will be anonymized, with only your initials and assigned number identified. As such, it will not be possible for anyone to identify, based on the data alone, who you are or where you live. Government authorities that supervise clinical studies, such as the Korean Ministry of Food and Drug Safety, may view the study records, but all such records will be managed with a password, making sure that only the investigators are aware of your name. Your name will not be disclosed in any academic report that publishes the study’s findings, either.

**18. Contact point in cases of impairment or injury related to the clinical study or if you wish to acquire additional information on study or the rights of study subjects**

If you wish to acquire additional information on the clinical study or the rights of study subjects, or report cases of impairment or injury related to the clinical study, you may contact the investigator.

This study has been approved by the IRB that has the obligation to protect the rights of study subjects and ensure their safety and welfare. If you have any questions regarding your rights as a study participant, please reach out to the individual in charge of IRB-related matters at our hospital (Tel. 02) 440-8107~8).

**19. Suspension of the participation in the study and reasons for such suspension**

During the course of the clinical study where it is required to take the drug three times a day for a total of 24 weeks, if the drug intake rate is lower than 80%, the participant shall be excluded from the study. Cases where a participant is deemed by the investigator as unfit for the study, too, shall be excluded from further participation.

The degree of side effects that serve as a cutoff point for stopping the study or bringing it to an end earlier than scheduled shall be determined based on the correlation with the treatment, the status of patients and the severity of the illness. If a severe side effect has occurred, the principal investigator shall make a decision based on the data of side effects in each individual. In addition, if deemed necessary by the principal investigator, participation in the study may be suspended or restricted without seeking the consent of the individual, but if new information is acquired that may affect ongoing participation in this study, an explanation shall be given to the individual or his/her representative in a timely manner.

This study is strictly based on your voluntary decision to participate. If you wish to withdraw your participation, you may do so at any point over the course of the study.

**20. Duration of data storage and the disposal of data**

Study records shall be stored by the investigator in a closed space with a lock. After the study is concluded, the records shall be moved to the IRB archive where it will be kept for three years. After the three-year period has expired, the records and documents shall be shredded to prevent the content from being disclosed. Any documents related to personal data shall be disposed of in accordance with Article 16 of the Enforcement Decree of the Personal Information Protection Act.

**21. Miscellaneous**

If you have received a briefing on the study thus far and have decided to take part in this study, please write your name and date on the consent form and give your signature in your own hand-writing. In the case that the study participant or his/her representative cannot read the information in the documents, consent form or information provided to study subjects, an observer shall be present throughout the entire process of seeking consent. One copy of the information sheet containing the information you have been given in the briefing and of the consent form you signed will be provided to you.

**Consent Form, ver. 1.4**

**Title of study**: The effect of Kami Guibi-tang for improving cognitive function in mild cognitive impairment patients : A randomized, double blind, placebo-controlled trial

**I hereby acknowledge that I was given sufficient explanation on content of this consent form, have thoroughly read and understood the content of the consent form and have received answers to my questions. I agree that I can ask the investigator at any point in time if I have questions regarding the study, and that my medical records will only be viewed for study purposes. I hereby voluntarily grant my consent to the participation in this clinical study.**

| **※ A copy of the signed document, ‘terms and conditions’ is to be provided.** | | | | |
| --- | --- | --- | --- | --- |
| Name of the clinical study participant |  | Signature |  | Date (Year/Month/Date) |
|  | | | | |
| Name of the guardian |  | Signature |  | Date (Year/Month/Date) |
| Legal representative Yes □ No □  Relationship with the study participant:  Detailed reasons: | | | | |
| Name of observer (if relevant) |  | Signature |  | Date (Year/Month/Date) |
|  | | | | |
| Name of the investigator who gave the explanation |  | Signature |  | Date (Year/Month/Date) |
